# High group B streptococcus carriage rates in pregnant women in a tertiary institution in Nigeria

**DOI:** 10.11604/pamj.2016.25.249.9433

**Published:** 2016-12-21

**Authors:** Charles John Elikwu, Oyinlola Oduyebo, Folasade Tolulope Ogunsola, Rose Ihuoma Anorlu, Christy Nene Okoromah, Brigitte König

**Affiliations:** 1 Department of Medical Microbiology & Parasitology, Ben Carson School of Medicine, Babcock University/Babcock University Teaching Hospitals, Ilisan-Remo, Ogun State, Nigeria; 2 Department of Medical Microbiology & Parasitology, Lagos University Teaching Hospital, Idi-Araba, Lagos, Nigeria; 3 Department of Medical Microbiology & Parasitology, College of Medicine, University of Lagos, Idi-Araba, Lagos, Nigeria; 4 Department of Obstetrics & Gynaecology, College of Medicine, University of Lagos, Idi-Araba, Lagos, Nigeria; 5 Department of Paediatrics and Child Health, College of Medicine, University of Lagos, Idi-Araba, Lagos, Nigeria; 6 Institut für Medizinische Mikrobiologie und Infektionsepidemiologie, Universitätsklinikum Leipzig, Germany

**Keywords:** Group B Streptococcus (GBS), carriage, pregnant women, GBS serotypes, GBS ST-17 lineage, meningitis, Lagos, Nigeria

## Abstract

**Introduction:**

In contrast to industrialized countries, until recently Group B Streptococcus (GBS) was infrequently reported in the developing world. This study was aimed at investigating the prevalence of GBS maternal colonization and to analyze the serotype distribution among the isolates.

**Methods:**

Vagino-rectal swabs collected from pregnant women were cultured for GBS using conventional media. Swabs were also taken from the mouths, ears and umbilical stumps of the neonates born to colonized mothers. Multiplex PCR and a conventional PCR to discern the *gbs2018*-ST-17 gene (specific for sequence type(ST)-17 clone) was performed to characterize the Group B streptococcus isolates.

**Results:**

A total of 300 pregnant women and 53 neonates were studied by culture but only 175 mothers by PCR. GBS was identified in four (6.8%) of 59 (19.7%) neonates of colonized mothers. Out of 175 mothers investigated by PCR, 112 (64%) were colonized. Serotype Ia (23.9%) was the most common among vagino-rectal isolates. Serotype II (71.4%) predominates among colonizing strain in newborns. A significant association between frequency of intercourse of > 2 per week and GBS carriage was found (t-test= 2.2; P value < 0.05).

**Conclusion:**

GBS carriage is high with low transmission. Strains that have been associated with GBS neonatal disease were reported, though in very low rates. Though none of the babies studied had invasive GBS disease, a more expansive study in the future will be required to establish if invasive GBS neonatal disease is uncommon in Nigeria.

## Introduction

Group B ß-haemolytic streptococci were first recorded as a cause of human infection in 1938 [1]. Since then, several reports have shown that Group B Streptococcus (GBS) is responsible for a substantial proportion of invasive diseases including pneumonia, septicaemia and meningitis in new-borns [2–4] as well as neonatal sepsis, neonatal death, pyelonephritis and premature rupture of membranes [5]. While this bacterium causes diseases in children, women, immunocompromised adult and the elderly [6, 7], serotype III strains have been significantly associated with meningitis in both early onset disease (EOD) and late onset disease (LOD) [8]. Since high genital inoculums at delivery, as detected by semi quantitative culture methods, significantly increase the likelihood of vertical transmission [9], infants, younger than 7 days, from heavily colonized women, are more likely to develop early-onset disease [10]. Its isolation from genital or lower gastrointestinal tracts of pregnant and non-pregnant women ranged from 5% to 40% [10]. However, when more than one appropriate site such as the lower vagina or the periurethral area or the rectum is sampled and selective broth media are utilized, the rate of colonization usually exceeds 20% [5, 10]. These variations in the reported prevalence of asymptomatic colonization are due to differences in the sites sampled, bacteriologic method for detection of the organism, and demographic differences in the populations studied.

Though the prevalence of GBS in pregnant women and non-pregnant women as well as neonatal invasive diseases are rarely reported [11] and 21% of women in labour carry GBS in their rectum and/or vagina [12], 12-25.8% rates in Australia [13] and 35% rates have been recorded in the USA [14]. In the UK, up to 20% of women carry GBS in the vagina or rectum without any associated symptoms [15, 16]. In the USA, it is the leading cause of neonatal disease since the 1970s [17–19]. In Nigeria, colonisation rates ranged from 6.6% to 20% [20–22]. Also, the perinatal mortality rates (PNMRs) of about 86 per 1000, one of the highest in the world, live births in the year 2000 [23] and PNMR of 78 per 1000 live births [24] have been indicated. This is in comparison to the perinatal mortality rate of 50 per 1000 live births in other developing regions of the world and 10 per 1000 live births in more industrialized countries. Consequently, while reducing childhood mortality levels by two-thirds by the year 2015 became one of the Millennium Development Goals (MDG-4) set by the United Nations [25] to substantially reduce perinatal deaths in high mortality countries [23, 26], perinatal mortality rate (PNMR) resulting from GBS infections has, also, become one of the key indicators of ill health [27]. Since there has been mounting reports that GBS is an important cause of neonatal sepsis in various parts of sub-Saharan Africa for over a decade [28–30], it is, therefore, pertinent to investigate the role of GBS in neonatal disease in Nigeria. This study was, therefore, designed to determine the vagino-rectal carriage of GBS colonization in pregnant women, to compare rates of microbial detection between specimen culturing and polymerase chain reactions (PCR) and to identify risk factors associated with GBS colonization as well as distribution of serotypes among isolates from pregnant women and their newborns.

## Methods

**Study area and population:** Participants for this study were recruited from the antenatal clinic of the Lagos University Teaching Hospital, (LUTH) a 761-bed tertiary care facility located in Surulere, Lagos, South-West Nigeria. Molecular studies were done at the *Institut für Medizinische Mikrobiologie und Infektionsepidemiologie, Universitätsklinikum*, Leipzig, Germany.

**Study design/population:** This was a cross-sectional study conducted between December 2010 and October 2011 at the Lagos University Teaching Hospital. The study recruited pregnant women at gestational age of = 28 weeks, attending the antenatal clinic of LUTH and those who presented in labour at the delivery wards. Participants were investigated for GBS carriage rates and most common serotypes. The newborns of colonized mothers were also included in the study.

**Inclusion criteria/exclusion criteria:** All consenting pregnant women with gestation age = 28 weeks, parturient women and their newborns were recruited into the study. Women below 28 weeks gestation and who had received antibiotics within two weeks of presenting at the antenatal clinic were excluded from the study.

**Sample size determination:** The minimum sample size was determined by the formula [31]: N = Z^2^ pq/d^2^, where, N = Sample size; P = 0.2 (based on Prevalence rate of 20% reported in previous local study; q = 1-p; Z = 1.96 (critical value at 95% confidence level); d = Precision, usually 5%; N = (1.96)^2^ (0.2)(0.8)/(0.05) ^2^ = 244. A sample size of 300 was used to increase the validity of the study with an expectation that not more than 20% of participants would be excluded in data analysis due to improperly filled questionnaire or refusal to respond to questions.

**Sampling technique:** The fact that universal screening for GBS among pregnant women has not yet been adopted as a policy in our environment and that potential participants were at different gestational age at presentation, convenience sampling was employed to choose participants from the population of pregnant women who presented for antenatal care and labor.

**Ethics statement:** The study protocol was approved by the institutional review board of Lagos University Teaching Hospital, Lagos, Nigeria (IRB No. ADM/DCST/221). Informed consent was confirmed by the IRB.

**Specimen collection:** A set of vagino-rectal swab samples consisting of two swabs were taken. Each swab was first inserted into the vagina and then the rectum. The first swab was the regular sterile cotton swab taken from each of the 300 pregnant women. The second was eSwabs (Copan Diagnostics, USA) collected from only 175 of the pregnant women. In addition, sterile cotton swabs from the mouth, ear and umbilical stump were taken from newborns included in the study. The regular sterile cotton swab did not have transport media but the eSwabs did. The cotton swabs and eSwabs were refrigerated in the clinic and immediately transported to the laboratory on ice packs for analysis on the day of collection. Vaginal swabs were transferred direct to the laboratory within 5 minutes of collecting samples from the antenatal clinic.

**Storage and transport of GBS Isolates and eSwab samples:** All GBS isolates from mothers and babies were stored in 20% glycerol broth (Brain Heart infusion containing 20% pure glycerol) and peptone water for freezing of organism in -80°C for molecular studies. The eSwab samples (eSwab 480CE, LQ Amies, Copan Diagnostics, USA) were stored at -20°C and were later transported in line with standard practice to the Institutfür Medizinische Mikrobiologie, und Infektions epidemiologie, Universitat sklinikum, Leipzig, Germany, for PCR studies.

### Analysis of specimen

**Microbiology:** Cotton swab samples from pregnant mothers and neonates were inoculated into Todd Hewitt broth supplemented with 15 µg/ml nalidixic acid and 10 µg/ml colistin (Biomerieux, Germany) subcultured on Columbia agar to which 5% sheep blood has been added (Oxoid, United Kingdom) and on freshly prepared CHROMagar™ StrepB(CHROMagar, France) before being incubated at 37°C in ambient air for 24-48 h. The colonies on the solid media were presumptively identified as Group B Streptococcus if they were Gram-positive cocci, catalase-negative, and positive to CAMP test [32] as well as forming small mauve to pink colonies on CHROMagarStrepB.

**DNA extractions:** Total DNA extraction from overnight GBS isolates and eSwabs suspensions was carried out using the DNeasy Blood & Tissue Kits (Qiagen, Germany) according to the manufacturer´sinstructions.

**PCR assays:** Confirmatory conventional PCR, a multiplex PCR assay for the identification of capsular serotype genes and a conventional PCR to discern the gbs2018-ST-17 gene (specific for sequence type(ST)-17 clone) were performed on all GBS isolates from mothers and newborns as previously described [33–35]. Direct PCR was performed on eSwabs samples to detect GBS using GBS-specific primers as previously described [35]. Strict precautions to prevent carryover of amplified DNA were applied as previously described [36].

**Statistical analysis:** Data entry and analysis were done using Epi-Info software, version 3.5.1. August 2008. Frequency tables, charts and cross-tables were used to present the data. The Chi-square test and Fisher’s exact test were used to compare the two groups, while the difference in means between groups was assessed using student’s t-test. p values < 0.05 were considered statistically significant.

## Results

**Socio-demographic characteristics:** A total of 300 pregnant women and 53 neonates were enrolled in the study. For logistics reasons, only 175 eSwabs from mothers were studied by PCR. The mean age of the participants was 30.95 ± 4.36 years (SD) (range 18-45 years). Table 1 shows socio-demographic characteristics of the women. The frequency of intercourse was 1 to 7 times a week with a mean of 1.73 and SD of 0.95.

**Table 1:** Socio-demographic characteristics and GBS status of Respondents

Demographic	GBS carriage		Total	Chi square	P value
Age Group	Carriage	No carriage			
<30	19	87	106	5.4	0.069
>30	30	158	182		
Not indicated	10	2	12		
Marital					
Married	48	236	284	5.0	0.116
Single	1	0	1		
Not indicated	10	5	15		
Educational level					
None	0	2	2	0.8	0.839
Primary	1	4	5		
Secondary	9	55	64		
Tertiary	39	185	224		
Not indicated	2	3	5		
Occupation					
None	14	71	85	1.0	0.821
Professional	11	53	64		
Skilled	11	43	54		
Unskilled	13	78	91		
Not indicated	4	2	6		
Parity					
1	18	82	100	1.7	0.645
2	31	164	195		
Not indicated	2	3	5		
Previous bad Pregnancy outcomes					
Yes	16	85	101	0.7	0.410
No	43	156	199		

**Table 2:** Association of age, parity and coital frequency with GBS carriage

	Carriage Mean (SD)	No carriage Mean (SD)	T test	P value
Age in years	31.2 (4.5)	30.9 (4.3)	0.4	0.685
Coital frequency per week	2.0 (1.2)	1.7 (0.9)	2.2	0.028
Parity (pregnancy)	2.2 (1.2)	2.3 (1.4)	0.7	0.502
Parity (abortion)	0.4 (0.8)	0.3 (0.6)	1.1	0.270

**GBS isolation and detection:** Fifty-nine (19.7%) out of 300 women studied by culture showed GBS-vaginal colonization, while 112 (64%) of 175 mothers (eSwab) investigated by PCR were colonized. The gene sequencing done on the randomly selected eSwab PCR products yielded a 97% match with the CAMP factor (cfb) gene sequence of Streptococcus agalactiae strain GDzl (accession number: GU217532.1). GBS was isolated from four (6.8%) neonates born to mothers with GBS-vaginal colonization and from seven (13.21%) neonates born to mothers in whom GBS was not cultured from vaginal swabs. All isolates were confirmed GBS by a conventional PCR.

**Figure 1:**
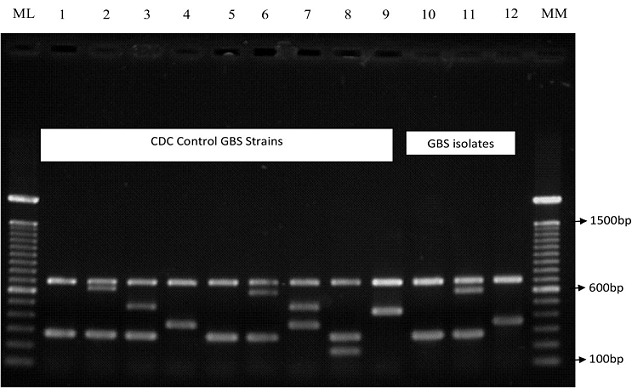
Gel electrophoresis identification of GBS capsular serotypes (cps) genes. Lane 1(serotype Ia reference strain); Lane 2 (serotype Ib reference strain); Lane 3 (serotype II reference strain); Lane 4 (serotype III reference strain); Lane5 (serotype IV reference strain); Lane 6 (serotype V reference strain); Lane 7 (serotype serotype VI reference strain); Lane 8 (serotype VII reference strain); Lane 9 (serotype VIII reference strain); Lane 10 (serotype Ia isolate); Lane 11 (serotype V isolate); Lane 12 (serotype III isolate); Lanes ML/MM (100bp molecular ladders)

**Figure 2:**
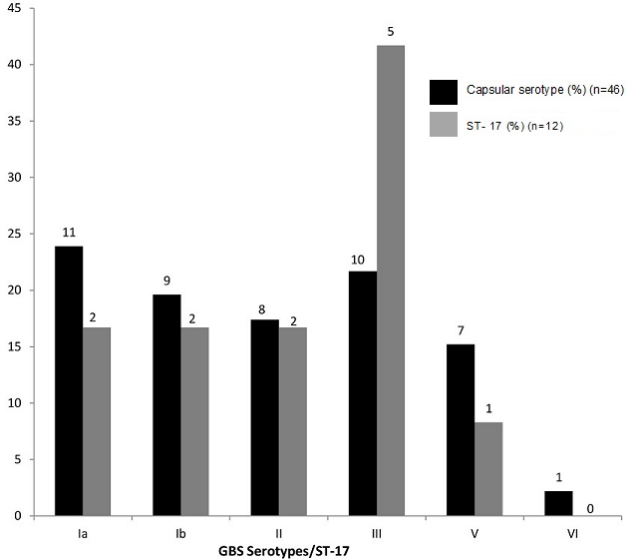
ST- 17 distributions among maternal GBS Serotypes

**Figure 3:**
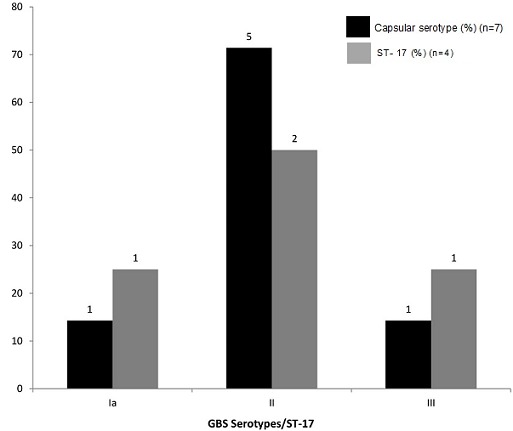
ST- 17 distributions among neonatal GBS Serotypes

**Serotype distribution/ST-17 clone among GBS Isolates:** Of the 59 vagino-rectal colonizing isolates, only 46 (78%) as well as seven of 11 colonizing strains in the newborns were available for molecular capsular typing and gbs2018-ST-17 gene determination. Figure 1 shows the characteristic bands pattern of the GBS serotypes distribution on agarose gel. CPS type Ia was the most common of the colonising strains in mothers (23.9%, n=11, Figure 2) while CPS type II was the most common GBS strain in newborns (n=5). Only one CPS type III occurred among colonising strains in the newborns. Four of the seven colonizing isolates in newborns, and 12 vagino-rectal colonizing isolates, were of the ST-17 lineage or clone. Among colonizing strains in newborns, this clone comprised of two strains of serotype II and one strain each of serotype Ia and III as shown in Figure 3.

**Risk factors for GBS colonization/Infection:** The association between risk factors and colonization of the maternal vagino-rectal region by GBS is summarized in Table 2 indicating a frequency of intercourse ≥ two times per week as a risk factor for GBS colonization.

## Discussion

In this study the rate obtained by culture only was much lower than the rate obtained by PCR, but falls in the range of carriage rates (6.6% to 20%) reported from previous studies in pregnant Nigerian women [30, 37–39]. The wide disparities between culture and PCR results could be attributed to the presence of nonviable GBS in the vaginal swabs or low bacterial load that could not be detected by culture but who’s DNA would be present for PCR amplification [40, 41]. Also, GBS detection by culture could also be inhibited by antibiotics and feminine hygiene products [40]. Using a culture method alone, some pregnant women colonized by GBS might be missed, especially in Nigeria where there are high rates of antibiotic misuse and abuse [42]. Despite the high colonisation rate in the present study, the low mother-to-baby transmission rate (6.8%) based on culture results in mothers during labour was much lower than the 28% transmission rate obtained from a similar study in the hospital in 1988 [43]. This may be due to widespread and discriminate use of penicillin to which GBS is still susceptible [42]. Also, it is a common obstetrics practice in Nigeria to wipe the vulva with antiseptic lotion during repetitive vagina examination during labour. This may be the reason why the high maternal carriage of GBS in this study did not translate to a correspondingly high rate of transmission to the newborns. In addition, none of the colonised babies developed invasive disease. This finding confirmed earlier observations that GBS is not a common cause of neonatal infection in Nigeria [30, 37, 43]. In recent times, studies from Kenya [23], South Africa [26, 29], Zimbabwe [27, 44] and Malawi [45] suggest that GBS is an important cause of neonatal sepsis in Africa, a study from south-eastern part of Nigeria reported a case of GBS neonatal sepsis from 33 septicaemic neonates screened [46].

In contrast to the study conducted in this hospital in 1988 in which the predominant strain isolated was serotype III (20/36; 55%) from both mothers and babies [43], this study showed that there are a variety of colonising serotypes and they are similar to serotypes circulating globally. Here, serotypes Ia and III were the predominant serotypes found in mothers (44.7%) in contrast to predominantly serotype II found in the babies (71.4%). These contrasting pictures suggested, perhaps, there has been a shift in circulating GBS serotypes. This range of serotypes distribution is similar to those found across the African subcontinent, though the most common serotypes may differ from country to country [28, 44, 45]. Also, GBS serotype II-ST-17 strains were rarely associated with invasive disease in neonates unlike their serotype III-ST-17 counterparts. This could be attributed to the fact that relative to serotype III strains, other serotypes showed reduced invasive potential [34, 47, 48]. In this study, there was a significant association between coital frequency of twice a week or more and GBS carriage (X^2^ = 2.2; p value < 0.05). This finding raises a number of questions. Is GBS sexually transmitted? Is there an association between GBS vaginal colonization and an elevated pH in the vaginal environment? However, a number of literature had reported this association [49–54]. Although, other predisposing factors such as preterm delivery, prolonged rupture of membrane and intra-amniotic infection [55, 56] that increases the baby’s risk of a GBS colonisation and infection were absent in all mothers studied, three women with positive GBS carriage had intrapartum fever.Prevention strategies ofGBS neonatal infection by identifying and prophylactically treating pregnant women, avoiding postpartum problems and early neonatal infections [56, 57] would be a worthy intervention.

## Conclusion

This study showed that GBS carriage in the studied population was high and transmission rate was low and indicated low rates of association between the serotypes and the diseases. Of significance, the study suggested a changing epidemiology of GBS which therefore requires regular surveillance for early detection of the introduction of more virulent strains that may signal the potential for invasive GBS disease among our neonates. Though, none of the babies studied had invasive GBS disease, a more expansive study in the future will be required to validate how uncommon the invasiveness of GBS neonatal disease is in Nigeria while prenatal screening of pregnant women at 35-37 weeks of gestation should be in view as efforts are being made towards the implementation of institutional and national antibiotic stewardship.

### What is known about this topic

- Group B *Streptococcus, GBS (Streptococcus agalactiae)* is a recognized and an important cause of maternal and neonatal mortality and morbidity in many parts of the world;
- Group B *Streptococcus* is one of the most frequent pathogens isolated from neonates with invasive bacterial disease.

### What this study adds

- This study indicate low rates of association between the serotypes and the diseases as none of the babies studied had invasive GBS disease;
- However, the study suggested a changing epidemiology of GBS which therefore requires regular surveillance for early detection of the introduction of more virulent strains that may signal the potential for invasive GBS disease among Nigerian neonates.
